# *NetMHCpan*, a Method for Quantitative Predictions of Peptide Binding to Any HLA-A and -B Locus Protein of Known Sequence

**DOI:** 10.1371/journal.pone.0000796

**Published:** 2007-08-29

**Authors:** Morten Nielsen, Claus Lundegaard, Thomas Blicher, Kasper Lamberth, Mikkel Harndahl, Sune Justesen, Gustav Røder, Bjoern Peters, Alessandro Sette, Ole Lund, Søren Buus

**Affiliations:** 1 Center for Biological Sequence Analysis, BioCentrum-DTU, Technical University of Denmark, Lyngby, Denmark; 2 Department of Experimental Immunology, Institute of Medical Microbiology and Immunology, University of Copenhagen, Copenhagen, Denmark; 3 La Jolla Institute for Allergy and Immunology, San Diego, California, United States of America; Federal University of Sao Paulo, Brazil

## Abstract

**Background:**

Binding of peptides to Major Histocompatibility Complex (MHC) molecules is the single most selective step in the recognition of pathogens by the cellular immune system. The human MHC class I system (HLA-I) is extremely polymorphic. The number of registered HLA-I molecules has now surpassed 1500. Characterizing the specificity of each separately would be a major undertaking.

**Principal Findings:**

Here, we have drawn on a large database of known peptide-HLA-I interactions to develop a bioinformatics method, which takes both peptide and HLA sequence information into account, and generates quantitative predictions of the affinity of any peptide-HLA-I interaction. Prospective experimental validation of peptides predicted to bind to previously untested HLA-I molecules, cross-validation, and retrospective prediction of known HIV immune epitopes and endogenous presented peptides, all successfully validate this method. We further demonstrate that the method can be applied to perform a clustering analysis of MHC specificities and suggest using this clustering to select particularly informative novel MHC molecules for future biochemical and functional analysis.

**Conclusions:**

Encompassing all HLA molecules, this high-throughput computational method lends itself to epitope searches that are not only genome- and pathogen-wide, but also HLA-wide. Thus, it offers a truly global analysis of immune responses supporting rational development of vaccines and immunotherapy. It also promises to provide new basic insights into HLA structure-function relationships. The method is available at http://www.cbs.dtu.dk/services/NetMHCpan.

## Introduction

Proteins are essential immune target structures. Being extremely diverse, they constitute unique imprints of their source organisms and provide-even at the peptide level-sufficient target identification and discrimination (reviewed in [1]). The cytotoxic T lymphocyte (CTL) arm of the T cell immune system represents a prime example of peptides being used as immune targets. CTL's are aimed at intracellular pathogens and obtain information on the intracellular environment of our cells through a series of cellular events involving HLA-I-mediated antigen processing and presentation of peptide epitopes derived from the intracellular protein metabolism, including that of intracellularly located pathogens (reviewed in [2]). A detailed description of how the immune system handles proteins and generates peptide could enable scientists and clinicians to analyze any protein of interest for the presence of potentially immunogenic CTL epitopes. Scanning entire proteomes computationally should further enable a rational approach to vaccine development, immunotherapy and diagnostics. Thus, candidate epitopes might be predicted from the various microbial genome projects, tumor vaccine candidates from mRNA expression profiling of tumors (“transcriptomes”) and auto-antigens from the human genome (reviewed in [1], [3]).

The single most selective event in antigen processing and presentation is that of peptide binding to HLA-I. It has been estimated that only 1 in 200 peptides will bind to a given MHC class I molecule with sufficient strength to elicit an immune response [2]. This makes it particularly important to establish accurate descriptions and predictions of peptide binding to HLA-I molecules [2]. It is not a simple task since the genes encoding HLA proteins are extremely polymorphic giving rise to many different peptide binding specificities being expressed in the human population. Sette and Sidney clustered HLA-I molecules into supertypes [4], [5] according to peptide binding specificities. Although the HLA-I supertype concept does reduce the complexity of the HLA-I system, there is still an unmet need to increase the coverage of HLA-I specificities as most existing HLA-I molecules have no or poorly characterized supertype relationships. Furthermore, at the present rate of discovery of HLA specificities, it would be a very demanding task to keep up with the increasing number of registered HLA molecules. Clearly, there is a need for a more efficient approach to analyze HLA-I specificities.

The analysis of HLA-I specificities have classically entailed the identification of peptide binding motifs (characterized primarily by the requirement for a few properly spaced and essential primary anchor residues) through pool sequencing of MHC eluted peptides [6] and/or the generation of a representative set of peptide binding data [7], [8]. Once such information has been obtained, the next step has been to generate peptide-binding predictions using either simple motif searches strategies [8] or complete statistical matrices representing the frequency of each amino acid in each position [9]–[13]. More recently, the growing amount of peptide-binding data has supported the generation of more sophisticated data-driven bioinformatics approaches including artificial neural networks, hidden Markov models, and support vector machines [14]–[20]. Artificial Neural Networks (ANN) are ideally suited to recognize non-linear patterns, which are believed to contribute to peptide-HLA-I interactions [15], [16], [21], [22]. In an ANN, information is trained and distributed into a computer network with an input layer, hidden layers and an output layer all connected in a given structure through weighted connections [23]. They are trained to recognize inputs (e.g. peptide sequences) associated with a given output (e.g. binding affinity). Once trained, the network should recognize the complicated input patterns compatible with binding. In a recent study, the ANN approach was found to be a highly efficient prediction mechanism for peptide-HLA-I interactions [24].

In general, HLA-I binding predictions depend on sufficient experimental data being available for the exact HLA-I molecule in question. Unfortunately, less than 10% of the 1500 [25] registered HLA-I proteins have been examined experimentally, and less than 5% have been characterized with more than 50 examples of peptide binders [26], [27]. Furthermore, focus has been towards the most prevalent Caucasians HLA-I molecules, which are not necessarily those prevalent among other populations, which are in more urgent need of new vaccine initiatives. By way of example, only two of the six HLA-A alleles, which are found with phenotype frequencies above 10% in Sub-Saharan African populations, are found above the 2–4% level in Caucasians; only three out of seven HLA-A alleles, which are found with phenotype frequencies above 10% in South-East Asian populations, are found above the 1% level in Caucasians; only three out of five HLA-A alleles, which are found with phenotype frequencies above 10% in South-American populations, are found above the 1% level in Caucasians etc. [28]. To overcome this problem, several (frequently computer intensive) prediction algorithms have been proposed using the three dimensional structure of the MHC molecule, and empirical or semi-empirical force fields, to estimate the peptide-HLA-I binding affinity [29]–[32]. Obviously, to extend this approach beyond the 17 HLA-I molecules currently solved at the structural level requires some kind of structural modeling [33]. Searching for alternative solutions, we here propose a novel method, *NetMHCpan*, exploiting both peptide and primary HLA sequence as input information for ANN-driven predictions pooling all available data and at the same time incorporate all HLA specificities. The method is successfully demonstrated to predict the affinity of interaction of any peptide with any human HLA-A or HLA-B molecule i.e. the method is pan-specific. Where other groups earlier have suggested similar prediction strategies to span limited regions of the HLA diversity [34]–[36], to the best of our knowledge, this is the largest database of HLA binding events ever used for this purpose, and the first report describing predictors applicable to a complete analysis of all HLA-A and -B specificities.

## Results

A large set of quantitative peptide-HLA binding data was used as input to train the *NetMHCpan* method. Both peptide and HLA primary sequences would subsequently be used as input for the method, and as output one should retrieve the predicted peptide-HLA-I binding affinity (for details see Materials and Methods).

### Experimental validation

**Figure 1:**
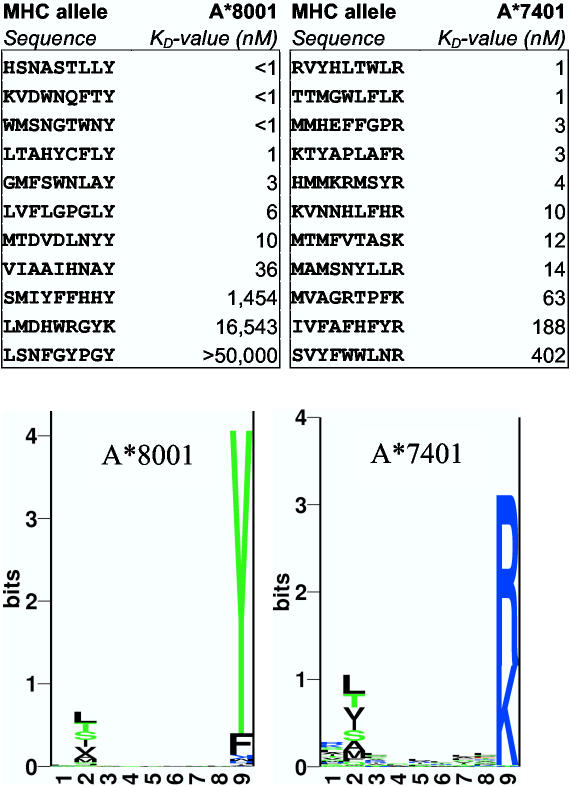
Prospective validation using hitherto uncharacterized HLA molecules. The upper figure gives the IC50 binding values for the sets of peptides identified by the *NetMHCpan* method to bind two hitherto uncharacterized HLA-A*8001, and HLA-A*7401 molecules. The peptides were selected as described in the text. 86% of the tested peptides bind stronger than 500 nM. The lower figure shows a Kullback-Leibler [52] logo visualization of the HLA binding motifs as predicted by the *NetMHCpan* method. Peptide binders used to generate the logos for each HLA molecule were selected from a pool of 500,000 random natural nonamers using the *NetMHCpan* method with a binding threshold of 500 nM. The logos were generated with the logo program of Schneider and Stephens [53]. Note that the binding motifs visualized in the logo plot are estimated from a set of approximately 5000 predicted binders, whereas the validated peptides only make up of the top 0.2%.

A prospective validation was performed using *NetMHCpan* to identify peptides, which would bind to HLA molecules that specificity-wise were unknown to us. For each HLA molecule, the binding affinity was predicted for a set of 500,000 random nonameric peptides of pathogenic, or human, origin. Only peptides predicted to bind with an affinity stronger than 50 nM were selected, and from this set of predicted binders, a subset of 10–15 peptides with low mutual sequence similarity (i.e. avoiding redundancy) was selected. These peptides were then tested for binding to the relevant HLA molecule in an *in vitro* binding assay [37]. More than 86% of the predictions were experimentally confirmed as binders with K_D_ values below 500 nM (many peptides bound with affinities better than 5 nM, see Figure 1). Thus, the pan-specific prediction approach was capable of extracting HLA sequence information and correctly relating this to peptide binding even in the absence of any data for the specific query HLA molecule.

### Leave-one-out validation

The ultimate validation of the predictive performance of the pan-specific approach is obtained by using the NetMHCpan method to identify peptide binders for MHC molecules that are specificity-wise unknown. This we have shown above for two alleles HLA. As another evaluation of the predictive performance of the pan-specific approach we performed a simulated “blind” leave-one-out validation. Here, we trained networks using all data for the relevant loci, HLA-A or -B, except the data for the molecule in question (i.e. a “leave-one-out” validation, here after refereed to as *Pan*). This was done for all HLA molecules represented in the data set. Thus, in this evaluation, no peptide-HLA binding data from the validation set was included in the training of the pan-specific predictor. For comparison, predictions were also trained solely on peptide binding data (i.e. without considering HLA sequence information) and using conventional cross-validation (see Materials and Methods). For each allele under consideration, we trained three such conventional single allele cross-validated networks based on different sets of peptide binding data: (1) data from the exact HLA molecule in question (*Self*), (2) data from the most closely related HLA molecule as identified by similarity between the HLA sequences (*Neighbor*), and (3) data from a previously selected representative of the HLA supertype [5] (*Supertype*; clearly this comparison cannot include the representative itself). This leave-one-out experiment thus constitutes a highly rigorous validation of the pan-specific method. By performing the leave-one-out experiment of all 42 alleles included in the benchmark data set, we can validate the performance of the *NetMHCpan* method on 42 alleles with uncharacterized binding specificity.

**Figure 2:**
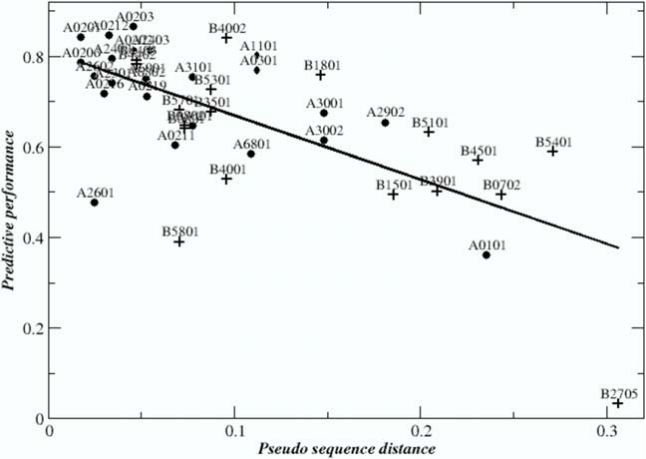
Predictive performance of the *NetMHCpan* method as a function of the distance to its nearest neighbor HLA allele. The nearest neighbor distance is estimated from the alignment score of the HLA pseudo sequences using the relation 
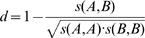
, where s(A,B) is the BLOSUM50 alignment score [47] between the pseudo sequences for alleles A and B, respectively. HLA-A alleles are shown as solid circles. HLA-B alleles are shown as +. The Pearson correlation coefficient between the pseudo sequence distance and the predictive performance for the 42 HLA alleles included in the plot is 0.67. Note, that the distance measure inherently assumes that all residues are equally important and independent of the pseudo sequence context. While this assumption is obviously inconsistent with the reality of primary anchors, it meets another essential requirement; it is simple and unbiased.

Some highlights of the “leave-one-out” analysis are shown in Table 1 (the complete data is given in Table S1). Perhaps not surprising, *Self* often performed better than *Pan*. However, it is noteworthy that all alleles, where *Pan* performed best, were characterized by very little data (57 to 141 peptide data points) being available. More pertinent for this work, however, *Pan* had a significantly higher predictive performance than both the *Neighbor and Supertype* methods (p<0.005 in both cases). Plotting the *Pan* performance against the distance between the query HLA and its nearest neighbor (as determined from the similarity between the two HLA sequences), it became apparent that the *Pan* predictor performed better when the query HLA molecule was represented by closely related HLA molecules (see Figure 2 and Table S2).

**Table 1:** Comparison of various validated predictors of peptide-HLA binding.

***(A)***						
*ANN*	A*0211	A*0212	A*0216	A*0219		
***Pan***	**0.60**	**0.85**	**0.72**	**0.71**		
***Self***	0.26	0.67	0.46	0.52		
***Neighbor***	0.49	0.74	0.56	0.56		
***Supertype***	0.49	0.74	0.56	0.65		
***# Data points***	141	113	57	137		
***(B)***						
***ANN***	A*2601	A*2602	B*5801	B*5701	B*4001	B*4402
**Pan**	0.48	**0.76**	0.39	0.68	0.53	**0.78**
**Self**	**0.80**	0.67	**0.84**	**0.83**	**0.82**	0.71
**Neighbor**	0.25	0.75	0.55	0.69	0.59	0.77
**Supertype**	NA	0.75	NA	0.69	NA	0.27
**# Data points**	1032	76	1340	59	1257	119
***(C)***						
***ANN***	B*2705	A*0101	B*0702			
***Pan***	0.03	0.36	0.49			
***Self***	**0.82**	**0.88**	**0.88**			
***Neighbor***	0.21	0.27	0.53			
***Supertype***	NA	NA	NA			
***# Data points***	1257	1213	1572			
***(D)***						
***ANN (locus average)***	***HLA-A***	***HLA-B***				
***Pan***	**0.75**	0.69				
***Self***	0.73	**0.78**				
***Neighbor***	0.57	0.61				
***Supertype***	0.57	0.45				
***# Data points***	26503	10881				

Experimental peptide-HLA binding data was used to develop artificial neural networks. The numbers given in the table are the Pearson correlation coefficients between the logarithmically transformed predicted binding affinities (K_D _values) and logarithm transformed observed binding affinities (K_D_ values). In bold are highlighted the maximum values in each column. (A) illustrates how poorly populated HLA molecules are more accurately predicted by the pan-specific leave-one molecule-out (*Pan*) predictor than by any of the conventional single allele predictors, even those generated using the data for the molecule in question. (B) illustrates that the pan-specific *Pan* predictor is only accurate when it has been trained on well-populated and relevant data. (C) illustrates that the pan-specific *Pan* predictor is inaccurate when no relevant data was included in the training sets. (D) illustrates the average performance for the HLA-A and –B locus molecules including random negative data. Note, only non-supertype representative alleles are included in the average. The predictors are *Pan*: the pan-specific ANN trained on data emanate from all members of the locus in question (i.e. HLA-A or –B) *except* for the member in question; *Self*: The most stringent comparison would be to use cross-validated ANN generated using data from the member in question, *Neighbor*: In the absence of self data, the next best alternatives would be to use cross-validated ANN generated using data from the most closely related member by BLOSUM comparison of the HLA-A (-or-B) pseudo-sequences, or *Supertype*: use cross-validated ANN generated using data from the member representing the supertype.

Examples of how HLA molecules, which are sparsely populated in terms of available peptide binding data, can be represented by related and well-populated HLA molecules is provided in Table 1A. Here, the performances of *Pan* are much better than those of the corresponding *Self*. In agreement, there are very few peptide binding data points (between 51 and 141 data point) for these HLA-A molecules, however, in total there are more than 11,000 data points for closely related HLA-A*02xx molecules. How sparsely populated HLA molecules cannot serve as HLA representatives is demonstrated in Table 1B. For HLA-A*2601 the *Pan* method has a much lower performance than the *Self*-method, whereas the converse is true for the closely related HLA-A*2602. This is in agreement with the fact that HLA*2601 is well populated with peptide binding data, whereas HLA-A*2602 is not. There is thus sufficient HLA-A*2601 data to represent HLA-A*2602, but not vice versa. A similar phenomenon can be observed for HLA-B*5801 vs. HLA-B*5701 and HLA-B*4001 versus HLA-B*4402. As shown in Table 1C, the HLA-B*2705 *Self*-performance is excellent, whereas the *Pan*, as well as *Neighbor*, performances are appalling. This is in agreement with the amount of data available; there are 1251 self-data points, but no clear representative of HLA-B*2705 (the difference in HLA sequence to the nearest neighbor is very high, see Figure 2 and Table S2). Similar, although less dramatic, observations are made for HLA-A*0101, and HLA-B*0702. Finally, HLA-A*6801 provides an example of how the *Pan*-networks avoids completely misleading *Neighbor* predictions (see Table S1). For HLA-A*6801, the nearest neighbor is HLA-A*6802, however, using the HLA-A*6802 predictor as HLA-A*6801 representative had a very poor predictive performance of −0.04. In contrast, the predictive performance of the *Pan*-networks for HLA-A*6801 is 0.62.

A summary of the leave-one-out experiment is given in Table 1D. For the HLA-A locus molecules, the *Pan* approach performed slightly better than *Self* and much better than *Neighbor* or *Supertype* (P<0.001), thus providing strong unbiased overall support for the pan-specific approach. For the HLA-B locus molecules, the *Pan* approach performed slightly poorer than *Self*, but still significantly better than both the *Neighbor*, and *Supertype* approaches (P<0.005). The performance difference between HLA-A and -B locus molecules is most likely the result of the more limited amount of available HLA-B data trying to cover an even greater span of sequence and binding motif diversities (i.e. see Figure 2, and the fact that 7 HLA-B supertypes are defined as compared to 5 for the HLA-A locus).

### The final NetMHCpan predictors

Often small data sets contain a strong bias for both the negative and positive data since the data was selected to fit some predicted binding motif. One way to lower a potential bias in the negative data set is to add random data with assumed weak binding affinity values [18]. For HLA it is a reasonable assumption that randomly chosen peptides will be non-binders, and the ANN methodology is reasonably robust against the occasional error introduced. Thus, for the remaining work, we added 100 random peptides to all data sets. This did indeed improve all the predictions that depended upon sparsely populated HLA representatives (e.g. *Pan* predictions for HLA-A*2601 and HLA-B*5801). The predictive performance for the leave-one-out pan-specific predictors trained including added random negative data is shown in Table 2.

**Table 2:** Performance for the different alleles in terms of the Pearsons correlation for the “leave-one-out” experiment with added random negatives.

(A) Predictors of HLA-A locus molecules (with random negatives)							
	*Pan*	*Self*	*Neighbor*		*Supertype*		Count
**A0101**	0.46	0.88	0.26	A1101		A1	1213
**A0201**	0.87	0.89	0.82	A0206		A2	3876
**A0202**	0.81	0.81	0.74	A0203	0.76	A2	1447
**A0203**	0.87	0.89	0.83	A0202	0.82	A2	2046
**A0206**	0.79	0.82	0.76	A0201	0.76	A2	2055
**A0211**	0.63	0.39	0.47	A0201	0.47	A2	141
**A0212**	0.85	0.59	0.73	A0201	0.73	A2	113
**A0216**	0.76	0.31	0.52	A0201	0.52	A2	57
**A0219**	0.75	0.57	0.59	A0212	0.61	A2	137
**A0301**	0.79	0.84	0.76	A1101		A3	2488
**A1101**	0.84	0.87	0.80	A0301	0.80	A3	2247
**A2301**	0.77	0.71	0.76	A2402	0.58	A24	167
**A2402**	0.81	0.85	0.78	A2301	0.71	A24	418
**A2403**	0.83	0.84	0.82	A2402		A24	321
**A2601**	0.69	0.79	0.53	A2602		A26	1032
**A2602**	0.71	0.69	0.70	A2601	0.70	A26	76
**A2902**	0.69	0.86	0.07	A3101	0.53	A3	160
**A3001**	0.68	0.82	−0.11	A3002	0.68	A3	931
**A3002**	0.65	0.64	0.37	A3001	0.36	A1	92
**A3101**	0.77	0.84	0.62	A3301	0.53	A3	2123
**A3301**	0.66	0.76	0.56	A3101	0.09	A3	1140
**A6801**	0.62	0.80	−0.05	A6802	0.28	A3	1141
**A6802**	0.74	0.78	0.60	A6901	0.31	A2	1434
**A6901**	0.76	0.81	0.72	A6802	0.62	A2	1648
**Ave**	0.74	0.75	0.57				
**Ave ex supertypes**	0.75	0.73	0.57		0.57	Sum	26503
**(B) Predictors of HLA-B locus molecules (with random negatives)**							
	***Pan***	***Self***	***Neighbor***		***Supertype***		**Count**
**B0702**	0.55	0.88	0.53	B0801		B7	1572
**B0801**	0.62	0.75	0.53	B0802		B8	812
**B0802**	0.59	0.86	0.76	B0801	0.76	B8	724
**B1501**	0.41	0.83	0.37	B3501		B62	1284
**B1801**	0.76	0.85	0.30	B3501	0.28	B62	290
**B2705**	0.05	0.82	0.15	B4002		B27	1257
**B3501**	0.68	0.79	0.63	B5301	0.48	B7	982
**B3901**	0.48	0.71	0.24	B0801		B39	81
**B4001**	0.59	0.82	0.55	B4002		B44	1257
**B4002**	0.82	0.75	0.68	B4001	0.68	B44	118
**B4402**	0.80	0.70	0.77	B4403	0.29	B44	119
**B4403**	0.79	0.74	0.70	B4402	0.43	B44	119
**B4501**	0.54	0.73	0.50	B4402	0.12	B44	114
**B5101**	0.58	0.79	0.57	B5301	0.40	B7	244
**B5301**	0.75	0.79	0.69	B3501	0.43	B7	254
**B5401**	0.57	0.80	0.36	B0702	0.36	B7	255
**B5701**	0.68	0.72	0.69	B5801	0.69	B58	59
**B5801**	0.45	0.85	0.66	B5701		B58	1340
**Ave**	0.59	0.79	0.54				
**Ave ex supertypes**	0.69	0.78	0.61		0.45	Sum	10881

Performance values for the “leave-one-out” experiment with added random negatives. (A) shows the performance for the 24 HLA-A alleles, and (B) the performance for the 18 HLA-B alleles. The first column gives the allele name, the following columns the performance of the *Pan, Self, Neighbor*, and *Supertype* methods, respectively, as explained in the text. After the *Neighbor* and *Supertype* performance values is shown the neighbor allele name and supertype association, respectively. Note, that the supertype performance is only stated for the non-supertype representing alleles. The final column gives the number of peptide data for each allele.

The final HLA-A and HLA-B pan-specific ANNs were trained on the complete datasets in a fivefold cross-validated manner on the *complete* data set abandoning the leave-one-out approach (see Materials and Methods). The Pearson correlation [38] for each HLA molecule was compared to that of the corresponding *Self*-networks. As illustrated in Table 3, the two approaches had comparable predictive performance. As the pan-specific neural network method demonstrates ability to encompass all HLA-A and HLA-B molecules, we denote the final pan-specific methods, *NetMHC-panA*, and *NetMHCpanB*, respectively.

**Table 3:** Performance of the pan-specific binding predictors.

*ANN*	*HLA-A*	*HLA-B*
***NetMHCpan***	0.77	0.77
**Self**	0.75	0.79

The average performance per locus of the pan-specific *NetMHCpanA* and *–panB* predictors vs. single allele specific ANN's trained using only data from available self-HLA molecules. Training and validation is done in a conventional cross-validated manner as described in Materials and methods with added random natural negative peptides.

We can estimate the sensitivity and specificity of the *NetMHCpan* method from the predictions of the 37,384 peptide data included in the benchmark. For a classification threshold of 500 nM, we find that the method has a specificity of 0.95, and a sensitivity of 0.74. Further, we find that 83% of the predicted binders are indeed experimentally verified binders. A complete table describing the relation between sensitivity and specificity is given in Table S3.

### Identification of HLA supertypes

**Figure 3:**
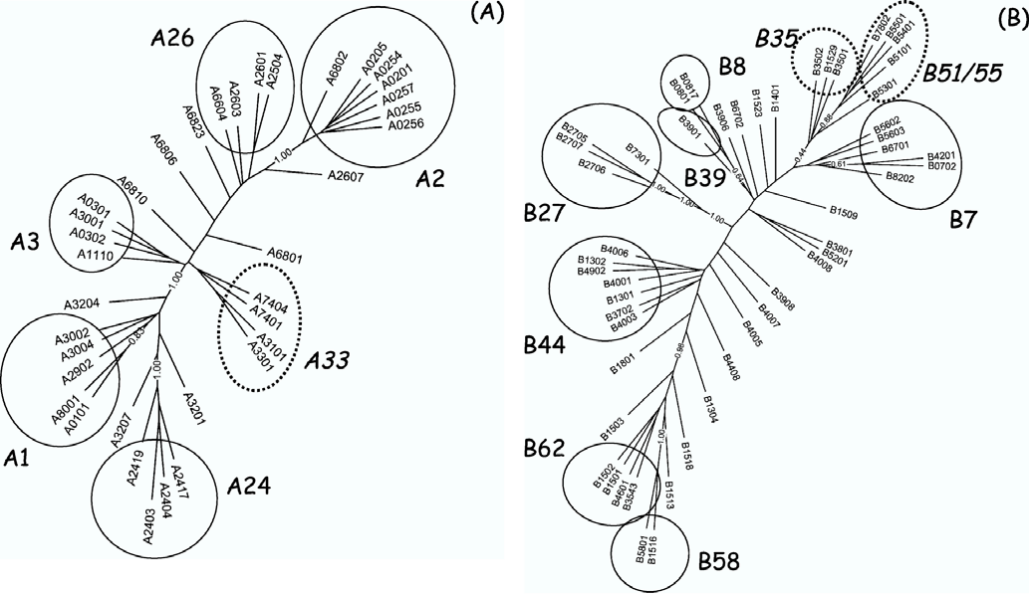
HLA clustering from *NetMHCpan* predictions. The left hand panel shows the clustering for 36 representative HLA-A alleles, and the right hand panel the clustering for 51 representatives HLA-B alleles. The trees are generated using the neighbor-joining algorithm from HLA distance matrices as described in the text. The 12 common supertypes are highlighted in full line circles. The proposed novel (sub)-supertypes are highlighted in dotted circles.

The pan-specific approach relies on the ability of the neural networks to capture general features of the relationship between peptides and HLA sequences, and interpret these in terms of binding affinity. Having demonstrated the predictive strength of the approach to identify the binding motif of uncharacterized HLA molecules, we now used the pan-specific ANNs to cluster HLA molecules according to predicted peptide binding specificity. Pruned HLA distance trees were calculated as described in Materials and Methods. Figure 3A depicts a tree including 36 representatives of the currently known HLA-A molecules, and Figure 3B a tree including 51 representatives of the known HLA-B molecules. The overall structure of the two new trees is in accordance with the supertype clustering proposed earlier by Sette and Sidney [4] and later extended by Lund et al, [5] according to which the HLA-A locus consists of five major supertypes A1, A2, A3, A24, and A26, and the HLA-B locus of seven major supertypes, B7, B8, B27, B39, B44, B58, and B62. However, the present analysis includes all known polymorphic HLA-A and -B molecules and suggests the existence of novel HLA supertypes, such as B51/B55, B35 (both split from B7), and A33, with specificities different from those described by previously defined HLA supertypes. Note also the assignment of the A*3001 molecule in the HLA-A tree. The A*3001 molecule has been variously clustered; by some to A3 [39], by others to A24 [4], and recently to A1 [5]. By the present analysis, it should belong to the A3 supertype. Reassuringly, this has subsequently been confirmed experimentally (Lamberth et al, manuscript in preparation).

### Identifying endogenously presented peptides

The NetMHCpan method was further validated using a large set of HLA ligand data. Nonamer HLA ligand data restricted to HLA-A and HLA-B alleles not included in the training data of the *NetMHCpan* method were downloaded from the SYFPEITHI database [11]. This set consists of 326 MHC ligands restricted to 43 different HLA-A and HLA-B alleles. For every peptide, the source protein was found in the SwissProt database [40]. If more than one source protein was possible, the longest protein was chosen. All nonameric peptides contained in the source protein sequences, except the annotated HLA ligand were taken as negative peptides. For each protein-HLA ligand pair the predictive performance was estimated as the percent rank of the HLA ligand among all nonamer peptides in the protein sequence. Performing this ranks calculation for all the 326 HLA ligands, we find a median rank of 1.4%. For half of the protein sequences, the HLA ligand is thus found within the top 1.4% highest scoring peptides. In a protein of size 300 amino acids, the HLA ligand will thus on average be ranked 4. The mean rank is 4.4%. These results demonstrate the predictive power of the pan-specific method to perform accurate predictions also for HLA alleles *not* included in the training.

### Predicting known HIV immunogens

As a final independent validation of the *NetMHC* approach, we analyzed all CTL nonamer epitopes reported with full HLA annotation in the Los Alamos HIV database (www.hiv.lanl.gov)[41]. This dataset contains 182 epitopes covering 49 HLA molecules (8 of these are of unknown HLA supertype assignment). The peptide-HLA binding affinity was predicted with *NetMHCpanA* or-*panB* using the annotated HLA molecule, and, when possible, with NetMHC (a previously reported HLA prediction tool available as www.cbs.dtu.dk/services/NetMHC) using the supertype representative. At a binding threshold of 500 nM, NetMHC identified 41% of the known epitopes, whereas the *NetMHCpanA* and -*panB* identified 52% (both approaches rejecting >98% of a random collection of nonamer peptides). Thus, the pan-specific approach recognized about 25% more known epitopes than an HLA supertype based approach.

## Discussion

Predictions of T cell epitopes have the potential to provide important information for rational research and development of vaccines and immunotherapies (reviewed in [1], [42]). Being computational, these tools enable a rapid and complete genomics analysis of all available pathogen isolates. Unfortunately, at this time they only cover a few of the many HLA specificities found in human populations. The method proposed here offers a complete incorporation of all human HLA specificities thereby covering a significant aspect of human immune diversity. Several groups have tried to develop methods for predicting which peptides will bind to a given HLA molecule [10], [14]–[20], [43], [44]. All such efforts have faced the problems of the limited amounts (or lack) of data available for most of the different HLA molecules present in the human population. Here, we report a pan-specific approach overcoming the problems of lacking specific binding data during the methods development. The major advantage of the pan-specific approach is that it predicts the binding of any peptide to any present and future HLA molecule, even in absence of data specific for the query HLA molecule, whereas conventional data-driven prediction approaches are restricted to predict the binding of peptides solely to the particular HLA molecules included in the training. In the past, others have proposed to similar strategies to span limited regions of the HLA diversity [34]–[36]. However, this is to our knowledge the first time data sets of this size have been available to do a complete analysis of all HLA-A and -B specificities.

The large-scale leave-one-out experiment, covering 42 distinct HLA-A and HLA-B alleles, provided unbiased support of the validity of the pan-specific ANN approach. It suggests that a pan-specific approach-given that there is sufficient and representative data available-is preferable to conventional approaches using single-allele specific prediction methods as defined by nearest *Neighbor* or *Supertype* representation. The pan-specific method is even preferable to conventional *Self* single-allele approach in cases where only limited data is available (e.g. 5 out of 6 HLA-A2 molecules with only few peptide data). It stresses the importance of the availability of large and representative HLA binding data, and it suggests that the development of the next generations of improved pan-specific predictors can be optimized through targeted selection of peptides and HLA molecules for future data inclusion.

The HLA supertype concept proposed by Sette and co-workers [4] suggested an approach to reduce the complexity of the polymorphism of the HLA. Several groups have developed methods for prediction of “promiscuous” HLA binders within known HLA supertypes [35], [36], [45]. However, all these methods require prior knowledge about the HLA supertype relationship, which for most HLA molecules remain undefined. Further, even if the supertype relationship is known, peptides identified to bind to a representative HLA molecule within a supertype might not bind to one or several of the other members of the same supertype. At the population level, the pan-specific approach promises an alternative strategy to handle HLA polymorphism and improve coverage in vaccine design. Rather than including one or more peptides restricted to each of the HLA supertypes, one could use the pan-specific HLA predictors in conjunction with the HLA frequency distribution within an ethnic population in question to select epitopes that will provide the broadest possible population coverage. A computer simulation of such a strategy for HIV specific CTL epitope identification suggest that coverage could be improved from some 90% for a supertype representation strategy to almost 100% for a pan-specific strategy (data not shown). At the individual level, it is obvious that the ability to handle any HLA molecule that a given patient might have irrespective of the availability of specific data for a particular HLA haplotype in question is an enabling technology for individualized immunotherapy and diagnostics.

It is implicitly clear that the pan-specific approach relies on the ability of the neural networks to capture general features of the relationship between peptides and HLA sequences, and interpret these in term of binding affinity. Using a polymorphism-based definition of the pseudo sequence (see Materials and Methods), we were able to generate pan-specific predictors of comparable predictive performance to that of predictors defined using the structure-based definition (data not shown). This supports our contention that the pan-specific approach amounts to a virtually complete analysis of the structure-function relationship of the polymorphic HLA system. It remains to be seen whether a deconvolution of the pan-specific ANN can unlock such information.

Intriguingly, our pan-specific predictors were able to predict peptide binders of closely related primate MHC class I molecules. For six of the most common Chimpanzee alleles represented in the Immune Epitope Database [27], more than 55% of the experimentally verified nonamer peptide binders could be predicted while maintaining a specificity of >95% (data not shown). This suggests that the specificity of closely related primate MHC molecules overlaps extensively with that of HLA molecules as earlier proposed by Sidney and co-workers[46]. We are currently investigating whether the pan-specific predictors can be used to identify peptide binders for, and perhaps even identify supertype relationships of, non-human primate MHC molecules (Nielsen et al., manuscript in preparation).

The current versions the *NetMHCpanA* and -*panB* are publicly available at www.cbs.dtu.dk/services/NetMHCpan. We will continuously update this service as more data become available. In the future, we expect to expand it to cover HLA-C, HLA class II, as well as non-human MHC molecules.

## Materials and Methods

### Source data

Nonameric peptide-HLA binding data was obtained from two sources: peptide-HLA binding data recently published by Sette and coworkers [24], and data recently deposited at the IEDB by Buus and coworkers. In total, the data set consisted of 37,384 unique peptide-HLA interactions covering 24 HLA-A alleles and 18 HLA-B alleles (26503 and 10881 for the A and B alleles, respectively). Some 2600 peptide-HLA interactions were present multiple times and the average IC50 value was assigned as the peptide affinity. The majority of the peptides present in both dataset have very similar binding affinities, and 97.5% of those peptides share annotated binding affinities within a 1.5 fold range. Only less than 1% of the peptides differ with more than 10 folds in annotated binding affinity, and the two data sets are thus highly consistent. The number of peptide data for each of the 42 alleles is listed in Table S4.

### HLA pseudo sequence

**Figure 4:**
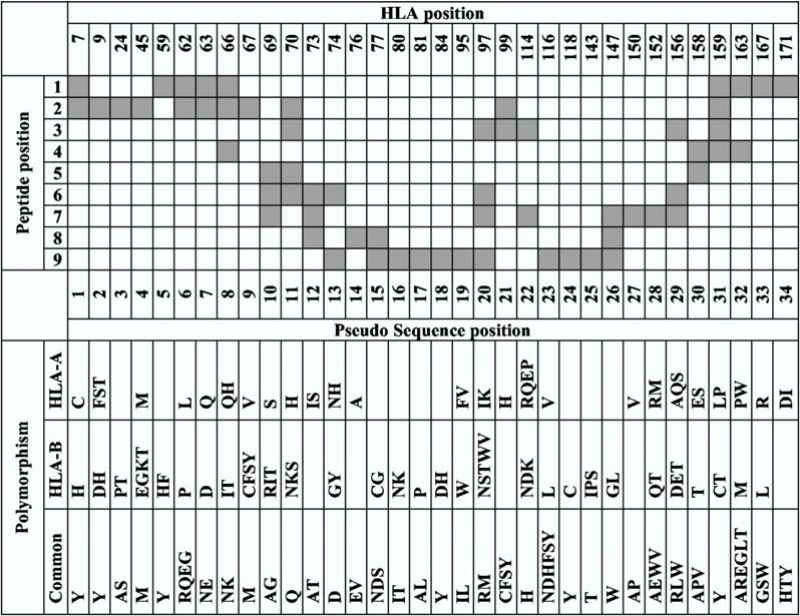
Definition of the HLA pseudo sequence. The upper part of the figure shows the residues of the HLA sequence estimated to be in contact with the peptide in the binding cleft. The columns give the HLA residue numbering according the IMGT nomenclature. The rows demonstrate the interactions with the nine peptides positions. Squares in grey outline the peptide positions estimated to have contact the corresponding HLA residue. The lower part of the figure shows the amino acid polymorphism at each position in the pseudo sequence, both those that are common for HLA-A and –B, and those that are unique for the HLA-A and HLA-B loci, respectively (as of February 2007).

The HLA sequence was encoded in terms of a pseudo-sequence consisting of amino acid residues in contact with the peptide. The contact residues are defined as being within 4.0 Å of the peptide in any of a representative set of HLA-A and -B structures with nonamer peptides. Only polymorphic residues from A, B, and C alleles were included giving rise to a pseudo-sequence consisting of 34 amino acid residues. Notice that due to multiple possible conformations, the central peptide residues could choose to interact with different subsets of residues in the binding groove. All such residues were included in the pseudo-sequence. The interaction map between the peptide and HLA sequence is given in Figure 4.

### Neural network training

Artificial neural networks were trained to quantitatively predict peptide-HLA binding. As input data, we used both peptide sequences and HLA primary sequence information, and as output data we used experimentally determined affinity data. The peptide data was obtained as described above. The primary HLA sequence information was obtained from the Anthony Nolan database (http://www.anthonynolan.org.uk/HIG/) and reduced to the 34 amino acid pseudo-sequence as described previously. The data was randomly split into five subsets, and five individual networks were trained each using 4/5 of the data to update the network weights and 1/5 to decide when to terminate the training (i.e. a five-fold cross-validation). Architectures with hidden neurons in the range 22 to 86 were tested, and the network with the highest prediction performance (lowest square error) on the test set was selected. The neural network architecture used was a conventional feed-forward network with one hidden layer and a single neuron output layer. A back-propagation procedure was used to update the weights in the network. For each data point, the input to the neural network is a sequence consisting of 43 peptide-HLA residues (9 from the peptide and 34 from the HLA), and as output the corresponding binding affinity was used. The binding affinity was log-transformed into the range between 0 and 1 as described by[15]. The input sequences were presented to the neural network in three distinct manners: a) conventional sparse encoding (i.e. is encoded by 19 zeros and a one), b) Blosum encoding, where each amino acid was encoded by the BLOSUM50 matrix score vector [47], and c) a mixture of the two, where the peptide was sparse encoded and the HLA pseudo sequence was Blosum encoded.

To estimate the predictive performance of the method, the leave-one-out experiment was conducted as briefly described here. Representing each HLA locus molecule, we trained a neural network ensemble using all available data for the relevant locus, excluding all data specific for the HLA allele in question. The network training was performed in a fivefold cross-validated manner as describe above resulting in an ensembles of in total 15 neural networks. The predicted affinity was then determined as the average of the 15 predictions in the neural network ensembles. In this benchmark calculation, the data for the allelic molecule in question was not involved in the training (and testing) of the method, and the performance was thus truly an unbiased test benchmark evaluation.

For the final NetMHCpan method, a conventional five-fold cross-validated training was performed. The pool of unique peptides was randomly split into five groups with all HLA binding data for a given peptide placed in the same group (in this way, no peptide can belong to more one group). The networks were trained as described above adapting the three different sequences encoding schemes, using 4/5 of the data to update the network weights and 1/5 to determine when to terminate the training.

### HLA distance trees

HLA distance trees were derived from correlations between predicted binding affinities. For each antigen, the binding affinity was predicted for a set of 10.000 random natural peptides using the NetMHCpan method. Next, the distance between any two alleles was defined, as D = 1-P_corr_, where P_corr_ is the Pearson correlation between two sets of predicted binding affinities. In this measure, two molecules that share a similar binding specificity will have a distance close to 0 whereas two molecules with unrelated binding specificities would have a distance close to 1. The HLA allele distance matrixes were calculated for 390 HLA-A alleles, and for 711 HLA-B alleles , and used the neighbor algorithm from the PHYLIP package, which implements the neighbor-joining algorithm of Saitou and Nei [48] to generate a HLA allele distance tree. To estimate the significance of the HLA distance tree, 100 such distance trees were generated using the bootstrap method [38]. The set of input trees were summarized in the form of a “greedy” consensus tree using proprietary software [49]. A greedy consensus tree uses a majority rule consensus tree to which all compatible bipartitions with frequencies below 50% have been added in order of descending frequency [50].

In order to visualize the HLA distance tree, only a subset of the leaves in the tree was displayed. The subset was selected in a Hobohm 1 like manner, where the alleles were clustered at a 0.95 distance level, and only a single allele from each cluster selected for display [51].

## Supporting Information

Table S1 Performance for the different alleles in terms of the Pearsons correlation for the “leave-one-out” experiment. Predictors of HLA-A and HLA-B locus molecules (without random negatives). (A) shows the performance for the 24 HLA-A alleles, and (B) the performance for the 18 HLA-B alleles. The first column gives the allele name, the following columns the performance of the Pan, Self, Neighbor, and Supertype methods, respectively, as explained in the text. After the Neighbor and Supertype performance values is shown the neighbor allele name and supertype association, respectively. Note, that the supertype performance is only stated for the non-supertype representing alleles. The final column gives the number of peptide data for each allele.
(0.11 MB DOC)

Table S2 Nearest neighbor identification for the 24 HLA-A and 18 HLA-B alleles. HLA-A and HLA-B allele nearest neighbor identification. (A) gives the nearest neighbor identification for the HLA-A alleles, (B) gives the nearest neighbor identification for the HLA-B alleles. The first column gives the allele name, the second column gives the Pan (leave-one-out pan-specific neural network) performance in terms of the Pearson correlation coefficient. The third and fourth columns give the allele name of the nearest neighbor and distance as determined from alignment of the pseudo sequences, the fifth column gives the predictive performance of the Neighbor method in terms of the Pearson correlation coefficient. Finally, the last column gives the number of data point available for the neighbor allele.
(0.08 MB DOC)

Table S3 Sensitivity and specificity relations for the NetMHCpan method. The table displays the sensitivity and specificity values at a classification threshold of 500 nM for the NetMHCpan method as estimated from the cross validated predictive performance for the 37,384 peptide data included in the benchmark data set. The number of binding peptides is 9665.
(0.04 MB DOC)

Table S4 The source data. The number of peptide binding data for each of the 24 HLA-A and 18 HLA-B molecules.
(0.05 MB DOC)
