# Supplementary material for: NetMHCpan, a Method for Quantitative Predictions of Peptide Binding to Any HLA-A and -B Locus Protein of Known Sequence
Source: PLoS One. 2007 Aug 29;2(8):e796. doi: 10.1371/journal.pone.0000796 (PMC1949492; doi:10.1371/journal.pone.0000796)
Supplement: Table S1 — Performance for the different alleles in terms of the Pearsons correlation for the “leave-one-out” experiment. Predictors of HLA-A and HLA-B locus molecules (without random negatives). (A) shows the performance for the 24 HLA-A alleles, and (B) the performance for the 18 HLA-B alleles. The first column gives the allele name, the following columns the performance of the Pan, Self, Neighbor, and Supertype methods, respectively, as explained in the text. After the Neighbor and Supertype performance values is shown the neighbor allele name and supertype association, respectively. Note, that the supertype performance is only stated for the non-supertype representing alleles. The final column gives the number of peptide data for each allele. (0.11 MB DOC) [file pone.0000796.s001.doc]

Table S1. Performance for the different alleles in terms of the Pearsons correlation for the “leave-one-out” experiment.

| **(A) Predictors of HLA-A locus molecules (without random negatives)** | | | | | | | |
| --- | --- | --- | --- | --- | --- | --- | --- |
|  | ***Pan*** | ***Self*** | ***Neighbor*** | | ***Supertype*** | | **N** |
| **A0101** | 0.36 | 0.88 | 0.27 | A1101 |  | A1 | 1213 |
| **A0201** | 0.84 | 0.90 | 0.81 | A0206 |  | A2 | 3876 |
| **A0202** | 0.81 | 0.81 | 0.75 | A0203 | 0.76 | A2 | 1447 |
| **A0203** | 0.87 | 0.89 | 0.80 | A0202 | 0.81 | A2 | 2046 |
| **A0206** | 0.79 | 0.83 | 0.76 | A0201 | 0.76 | A2 | 2055 |
| **A0211** | 0.60 | 0.26 | 0.49 | A0201 | 0.49 | A2 | 141 |
| **A0212** | 0.85 | 0.67 | 0.74 | A0201 | 0.74 | A2 | 113 |
| **A0216** | 0.72 | 0.46 | 0.56 | A0201 | 0.56 | A2 | 57 |
| **A0219** | 0.71 | 0.52 | 0.56 | A0212 | 0.65 | A2 | 137 |
| **A0301** | 0.77 | 0.84 | 0.77 | A1101 |  | A3 | 2488 |
| **A1101** | 0.80 | 0.88 | 0.80 | A0301 | 0.80 | A3 | 2247 |
| **A2301** | 0.74 | 0.76 | 0.73 | A2402 | 0.73 | A24 | 167 |
| **A2402** | 0.79 | 0.84 | 0.75 | A2301 |  | A24 | 418 |
| **A2403** | 0.82 | 0.84 | 0.81 | A2402 | 0.81 | A24 | 321 |
| **A2601** | 0.48 | 0.80 | 0.25 | A2602 |  | A26 | 1032 |
| **A2602** | 0.76 | 0.67 | 0.75 | A2601 | 0.81 | A26 | 76 |
| **A2902** | 0.65 | 0.86 | 0.08 | A3101 | 0.53 | A3 | 160 |
| **A3001** | 0.66 | 0.83 | 0.19 | A3002 | 0.68 | A3 | 931 |
| **A3002** | 0.62 | 0.67 | 0.34 | A3001 | 0.36 | A1 | 92 |
| **A3101** | 0.75 | 0.84 | 0.60 | A3301 | 0.52 | A3 | 2123 |
| **A3301** | 0.65 | 0.75 | 0.56 | A3101 | 0.08 | A3 | 1140 |
| **A6801** | 0.59 | 0.80 | -0.04 | A6802 | 0.28 | A3 | 1141 |
| **A6802** | 0.74 | 0.78 | 0.61 | A6901 | 0.31 | A2 | 1434 |
| **A6901** | 0.75 | 0.83 | 0.70 | A6802 | 0.63 | A2 | 1648 |
| **Ave** | 0.71 | 0.76 | 0.57 |  |  |  |  |
| **Ave ex sup** | 0.73 | 0.73 | 0.57 |  | 0.59 | Sum | 26503 |
|  |  |  |  |  |  |  |  |
| **(B) Predictors of HLA-B locus molecules (without random negatives)** | | | | | | | |
|  | ***Pan*** | ***Self*** | ***Neighbor*** | | ***Supertype*** | | **N** |
| **B0702** | 0.49 | 0.88 | 0.53 | B0801 |  | B7 | 1572 |
| **B0801** | 0.64 | 0.75 | 0.53 | B0802 |  | B8 | 812 |
| **B0802** | 0.65 | 0.87 | 0.77 | B0801 | 0.77 | B8 | 724 |
| **B1501** | 0.49 | 0.83 | 0.37 | B3501 |  | B62 | 1284 |
| **B1801** | 0.76 | 0.86 | 0.35 | B3501 | 0.32 | B62 | 290 |
| **B2705** | 0.03 | 0.82 | 0.21 | B4002 |  | B27 | 1257 |
| **B3501** | 0.68 | 0.78 | 0.61 | B5301 | 0.32 | B7 | 982 |
| **B3901** | 0.50 | 0.61 | 0.21 | B0801 |  | B39 | 81 |
| **B4001** | 0.53 | 0.82 | 0.59 | B4002 |  | B44 | 1257 |
| **B4002** | 0.84 | 0.83 | 0.70 | B4001 | 0.70 | B44 | 118 |
| **B4402** | 0.78 | 0.71 | 0.77 | B4403 | 0.27 | B44 | 119 |
| **B4403** | 0.79 | 0.82 | 0.78 | B4402 | 0.43 | B44 | 119 |
| **B4501** | 0.57 | 0.82 | 0.51 | B4402 | 0.13 | B44 | 114 |
| **B5101** | 0.63 | 0.76 | 0.55 | B5301 | 0.41 | B7 | 244 |
| **B5301** | 0.73 | 0.79 | 0.68 | B3501 | 0.44 | B7 | 254 |
| **B5401** | 0.59 | 0.82 | 0.37 | B0702 | 0.37 | B7 | 255 |
| **B5701** | 0.68 | 0.83 | 0.69 | B5801 | 0.69 | B58 | 59 |
| **B5801** | 0.39 | 0.84 | 0.55 | B5701 |  | B58 | 1340 |
| **Ave** | 0.60 | 0.80 | 0.54 |  |  |  |  |
| **Ave ex sup** | 0.70 | 0.81 | 0.62 |  | 0.44 | Sum | 10881 |

Predictors of HLA-A and HLA-B locus molecules (without random negatives). (A) shows the performance for the 24 HLA-A alleles, and (B) the performance for the 18 HLA-B alleles. The first column gives the allele name, the following columns the performance of the *Pan, Self, Neighbor*, and *Supertype* methods, respectively, as explained in the text. After the *Neighbor* and *Supertype* performance values is shown the neighbor allele name and supertype association, respectively. Note, that the supertype performance is only stated for the non-supertype representing alleles. The final column gives the number of peptide data for each allele.
