# Supplementary material for: NetMHCpan, a Method for Quantitative Predictions of Peptide Binding to Any HLA-A and -B Locus Protein of Known Sequence
Source: PLoS One. 2007 Aug 29;2(8):e796. doi: 10.1371/journal.pone.0000796 (PMC1949492; doi:10.1371/journal.pone.0000796)
Supplement: Table S2 — Nearest neighbor identification for the 24 HLA-A and 18 HLA-B alleles. HLA-A and HLA-B allele nearest neighbor identification. (A) gives the nearest neighbor identification for the HLA-A alleles, (B) gives the nearest neighbor identification for the HLA-B alleles. The first column gives the allele name, the second column gives the Pan (leave-one-out pan-specific neural network) performance in terms of the Pearson correlation coefficient. The third and fourth columns give the allele name of the nearest neighbor and distance as determined from alignment of the pseudo sequences, the fifth column gives the predictive performance of the Neighbor method in terms of the Pearson correlation coefficient. Finally, the last column gives the number of data point available for the neighbor allele. (0.08 MB DOC) [file pone.0000796.s002.doc]

Table S2. Nearest neighbor identification for the 24 HLA-A and 18 HLA-B alleles.

| (A) HLA-A allele nearest neighbor identification | | | | | |
| --- | --- | --- | --- | --- | --- |
| **Allele** | ***Pan*** | ***Neighbor*** | | | **N** |
|  |  | **Allele** | **Dist** | **Perf** |  |
| **A0101** | 0.36 | A1101 | 0.24 | 0.27 | 2247 |
| **A0201** | 0.84 | A0206 | 0.02 | 0.81 | 2055 |
| **A0202** | 0.81 | A0203 | 0.05 | 0.75 | 2046 |
| **A0203** | 0.87 | A0202 | 0.05 | 0.80 | 1447 |
| **A0206** | 0.79 | A0201 | 0.02 | 0.76 | 3876 |
| **A0211** | 0.60 | A0201 | 0.07 | 0.49 | 3876 |
| **A0212** | 0.85 | A0201 | 0.03 | 0.74 | 3876 |
| **A0216** | 0.72 | A0201 | 0.03 | 0.56 | 3876 |
| **A0219** | 0.71 | A0212 | 0.05 | 0.56 | 113 |
| **A0301** | 0.77 | A1101 | 0.11 | 0.77 | 2247 |
| **A1101** | 0.80 | A0301 | 0.11 | 0.80 | 2488 |
| **A2301** | 0.74 | A2402 | 0.03 | 0.73 | 418 |
| **A2402** | 0.79 | A2301 | 0.03 | 0.75 | 167 |
| **A2403** | 0.82 | A2402 | 0.05 | 0.81 | 418 |
| **A2601** | 0.48 | A2602 | 0.03 | 0.24 | 76 |
| **A2602** | 0.76 | A2601 | 0.03 | 0.75 | 1032 |
| **A2902** | 0.65 | A3101 | 0.18 | 0.08 | 2123 |
| **A3001** | 0.68 | A3002 | 0.15 | 0.19 | 92 |
| **A3002** | 0.62 | A3001 | 0.15 | 0.34 | 931 |
| **A3101** | 0.75 | A3301 | 0.08 | 0.60 | 1140 |
| **A3301** | 0.65 | A3101 | 0.08 | 0.56 | 2123 |
| **A6801** | 0.59 | A6802 | 0.11 | -0.04 | 1434 |
| **A6802** | 0.74 | A6901 | 0.05 | 0.61 | 1648 |
| **A6901** | 0.75 | A6802 | 0.05 | 0.70 | 1434 |
|  | | | | | |
| **(B) HLA-B allele nearest neighbor identification** | | | | | |
|  |  | ***Neighbor*** | | |  |
| ***Allele*** | ***Pan*** | ***Allele*** | ***Dist*** | ***Perf*** | ***NN*** |
| **B0702** | 0.49 | B0801 | 0.24 | 0.53 | 812 |
| **B0801** | 0.64 | B0802 | 0.07 | 0.53 | 724 |
| **B0802** | 0.65 | B0801 | 0.07 | 0.77 | 812 |
| **B1501** | 0.49 | B3501 | 0.19 | 0.36 | 982 |
| **B1801** | 0.76 | B3501 | 0.15 | 0.35 | 982 |
| **B2705** | 0.03 | B4002 | 0.31 | 0.21 | 118 |
| **B3501** | 0.68 | B5301 | 0.09 | 0.61 | 254 |
| **B3901** | 0.50 | B0801 | 0.21 | 0.21 | 812 |
| **B4001** | 0.53 | B4002 | 0.10 | 0.58 | 118 |
| **B4002** | 0.84 | B4001 | 0.10 | 0.70 | 1257 |
| **B4402** | 0.78 | B4403 | 0.05 | 0.77 | 119 |
| **B4403** | 0.79 | B4402 | 0.05 | 0.78 | 119 |
| **B4501** | 0.57 | B4402 | 0.23 | 0.50 | 119 |
| **B5101** | 0.63 | B5301 | 0.21 | 0.55 | 254 |
| **B5301** | 0.73 | B3501 | 0.09 | 0.68 | 982 |
| **B5401** | 0.59 | B0702 | 0.27 | 0.37 | 1572 |
| **B5701** | 0.68 | B5801 | 0.07 | 0.69 | 1340 |
| **B5801** | 0.39 | B5701 | 0.07 | 0.55 | 59 |

**HLA-A and HLA-B allele nearest neighbor identification. (A) gives the nearest neighbor identification for the HLA-A alleles, (B) gives the nearest neighbor identification for the HLA-B alleles. The first column gives the allele name, the second column gives the *Pan* (leave-one-out pan-specific neural network) performance in terms of the Pearson correlation coefficient. The third and fourth columns give the allele name of the nearest neighbor and distance as determined from alignment of the pseudo sequences, the fifth column gives the predictive performance of the *Neighbor* method in terms of the Pearson correlation coefficient. Finally, the last column gives the number of data point available for the neighbor allele.**
