# Supplementary material for: NetMHCpan, a Method for Quantitative Predictions of Peptide Binding to Any HLA-A and -B Locus Protein of Known Sequence
Source: PLoS One. 2007 Aug 29;2(8):e796. doi: 10.1371/journal.pone.0000796 (PMC1949492; doi:10.1371/journal.pone.0000796)
Supplement: Table S3 — Sensitivity and specificity relations for the NetMHCpan method. The table displays the sensitivity and specificity values at a classification threshold of 500 nM for the NetMHCpan method as estimated from the cross validated predictive performance for the 37,384 peptide data included in the benchmark data set. The number of binding peptides is 9665. (0.04 MB DOC) [file pone.0000796.s003.doc]

**Table S3. Sensitivity and specificity relations for the *NetMHCpan* method.**

| ***Sensitivity*** | ***Specificity*** | ***log50k*** | ***nM*** |
| --- | --- | --- | --- |
| 0.50 | 0.99 | 0.58 | 94 |
| 0.60 | 0.97 | 0.51 | 201 |
| 0.70 | 0.96 | 0.45 | 384 |
| 0.80 | 0.93 | 0.38 | 819 |
| 0.90 | 0.85 | 0.27 | 2693 |
| 0.95 | 0.76 | 0.18 | 7131 |

**The table displays the sensitivity and specificity values at a classification threshold of 500 nM for the *NetMHCpan* method as estimated from the cross validated predictive performance for the 37,384 peptide data included in the benchmark data set. The number of binding peptides is 9665.**
