# Supplementary material for: NetMHCpan, a Method for Quantitative Predictions of Peptide Binding to Any HLA-A and -B Locus Protein of Known Sequence
Source: PLoS One. 2007 Aug 29;2(8):e796. doi: 10.1371/journal.pone.0000796 (PMC1949492; doi:10.1371/journal.pone.0000796)
Supplement: Table S4 — The source data. The number of peptide binding data for each of the 24 HLA-A and 18 HLA-B molecules. (0.05 MB DOC) [file pone.0000796.s004.doc]

Table S4. The source data.

| ***HLA-A*** | ***#*** | ***HLA-B*** | ***#*** |
| --- | --- | --- | --- |
| A0101 | 1213 | B0702 | 1572 |
| A0201 | 3876 | B0801 | 812 |
| A0202 | 1447 | B0802 | 724 |
| A0203 | 2046 | B1501 | 1284 |
| A0206 | 2055 | B1801 | 290 |
| A0211 | 141 | B2705 | 1257 |
| A0212 | 113 | B3501 | 982 |
| A0216 | 57 | B3901 | 81 |
| A0219 | 137 | B4001 | 1257 |
| A0301 | 2488 | B4002 | 118 |
| A1101 | 2247 | B4402 | 119 |
| A2301 | 167 | B4403 | 119 |
| A2402 | 418 | B4501 | 114 |
| A2403 | 321 | B5101 | 244 |
| A2601 | 1032 | B5301 | 254 |
| A2602 | 76 | B5401 | 255 |
| A2902 | 160 | B5701 | 59 |
| A3001 | 931 | B5801 | 1340 |
| A3002 | 92 |  |  |
| A3101 | 2123 |  |  |
| A3301 | 1140 |  |  |
| A6801 | 1141 |  |  |
| A6802 | 1434 |  |  |
| A6901 | 1648 |  |  |
| **TOTAL** | **26503** | **TOTAL** | **10881** |

**The number of peptide binding data for each of the 24 HLA-A and 18 HLA-B molecules.**
